# Effect of Distracting Background Speech in an Auditory Brain–Computer Interface

**DOI:** 10.3390/brainsci11010039

**Published:** 2021-01-01

**Authors:** Álvaro Fernández-Rodríguez, Ricardo Ron-Angevin, Ernesto J. Sanz-Arigita, Antoine Parize, Juliette Esquirol, Alban Perrier, Simon Laur, Jean-Marc André, Véronique Lespinet-Najib, Liliana Garcia

**Affiliations:** 1 UMA-BCI Group, Departamento de Tecnología Electrónica, Universidad de Málaga, 29071 Malaga, Spain; afernandezrguez@uma.es; 2 Neuro and Aging and Human Cognition, INCIA-UMR 5287-CNRS, Université de Bordeaux, 33076 Bordeaux, France; ernesto.sanz-arigita@u-bordeaux.fr; 3 Laboratoire IMS, CNRS UMR 5218, Cognitive Team, Bordeaux INP-ENSC, 33400 Talence, France; aparize@ensc.fr (A.P.); jesquirol@ensc.fr (J.E.); aperrier004@ensc.fr (A.P.); slaur@ensc.fr (S.L.); jean-marc.andre@ensc.fr (J.M.-A.); veronique.lespinet@ensc.fr (V.L.-N.); liliana.audin@ims-bordeaux.fr (L.G.)

**Keywords:** brain–computer interface (BCI), event-related potential (ERP), auditory, distractor, workload

## Abstract

Studies so far have analyzed the effect of distractor stimuli in different types of brain–computer interface (BCI). However, the effect of a background speech has not been studied using an auditory event-related potential (ERP-BCI), a convenient option when the visual path cannot be adopted by users. Thus, the aim of the present work is to examine the impact of a background speech on selection performance and user workload in auditory BCI systems. Eleven participants tested three conditions: (i) auditory BCI control condition, (ii) auditory BCI with a background speech to ignore (non-attentional condition), and (iii) auditory BCI while the user has to pay attention to the background speech (attentional condition). The results demonstrated that, despite no significant differences in performance, shared attention to auditory BCI and background speech required a higher cognitive workload. In addition, the P300 target stimuli in the non-attentional condition were significantly higher than those in the attentional condition for several channels. The non-attentional condition was the only condition that showed significant differences in the amplitude of the P300 between target and non-target stimuli. The present study indicates that background speech, especially when it is attended to, is an important interference that should be avoided while using an auditory BCI.

## 1. Introduction

As a direct communication link between the brain and an external device without the presence of muscular activity, a brain–computer interface (BCI) relies on higher cognitive functions such as attention, working memory, and executive control to obtain an adequate performance [1,2,3,4,5]. Nowadays, several tools have been developed to bring this technology closer to a consumer device [6,7,8,9]. Ecological environments typical to the target population, as opposed to laboratory or clinical situations, are poorly controlled contexts where unexpected events and distractions may occur without real control by the users (e.g., external conversation, television, or people walking).

Important attentional resources are necessary to execute appropriate control of the BCI system. The allocation of these resources depends on both the user’s state and external conditions. Indeed, distractors, such as external stimuli not implicated in the control of the interface, or conflicting attentional demands could increase the workload on the user and directly affect the performance of the BCI. The effects of distractors on BCI controls have been studied previously using three types of electroencephalographic (EEG) signal: steady-state visual evoked potentials (SSVEP) [10], event-related (de)synchronization phenomena (ERD/ERS) [11,12,13], and event-related potentials (ERP) [14,15,16,17]. These studies have shown that distractors can affect the performance, the ERP waveform, or the subjective experience of the user.

The ERP signal has been extensively studied in the previous BCI literature, and several devices were proposed using this signal [18,19]. Auditory and visual BCI based on event-related potentials (ERP-BCI) have been developed to supplement motor deficiencies in amyotrophic lateral sclerosis patients [20]. However, auditory ERP-BCI is an alternative when the user’s visual pathway is affected and a visual BCI cannot be controlled [21,22,23,24,25]. Auditory ERP-BCIs use EEG-specific responses to the presentation of an uncommon auditory target stimulus among a set of possible options [26]. The most usual potential used as input for BCIs is the P300, a positive change in the EEG that occurs around 300 ms after the stimulus onset [27]. These BCIs are commonly based on the oddball paradigm: users receive a succession of auditory stimuli and they are asked to focus on one of them (an infrequent target stimulus) and to perform a simple mental task when it appears [17,25,28,29]. While this mental task is usually to keep count of how many times the target stimulus happens, other tasks have been studied, such as the mental repetition of the stimulus [30].

To the best of our knowledge, only three previous studies based on ERP-BCI have examined the effect of background: two were based on visual ERP-BCI and studied the effect of a background talk [15,16], and only one was based on auditory ERP-BCI, examining the effect of background music [17]. First, Käthner et al. [15] found a lower performance when participants had to pay attention to two concurrent stories presented over headphones (auditory attentional condition) compared with when they ignored them (auditory non-attentional condition). Additionally, the attentional condition presented a higher workload than the non-attentional condition at the beginning of the experimental session, but not at the end, when the scores were equal. The grand average of waveforms for the attentional condition was significantly smaller than that of the non-attentional condition. Later, Xu et al. [16] showed that requiring the user to listen to an audio story at different speeds decreased performance as the user workload increased (the faster the audio story, the greater the workload). They also found that the amplitude of the P300 component, in the parietal and occipital regions, was negatively related to the increase in difficulty of additional auditory tasks. Zhou et al. [17] presented the only paper to our knowledge that has assessed the effect of a background sound (instrumental music) while controlling an auditory ERP-BCI. They concluded that the background music made users more comfortable without a decrease in performance. These results are especially interesting since music could be considered as a type of distractor that, unlike speech in visual ERP-BCI, could improve the user experience.

In light of the above, the aim of the present study is to complete the spectrum of distractors for an auditory ERP-BCI. It investigates the effects of background speech on auditory BCI performance and workload and the impact of background speech with passive and active attention. In accordance with the effects of auditory distractors on a visual ERP-BCI, we hypothesized that background speech and an active attentional task would i) decrease performance, ii) increase user workload, and iii) decrease the amplitude of the P300 component.

## 2. Materials and Methods

### 2.1. Participants

Eleven native French speakers (mean: 21.09 years, range: 20–22, gender: 2 male) participated in the present study. None of the participants had previous experience in the use of auditory ERP-based BCIs. According to self-reports, none of the participants had any history of neurological or psychiatric illness. In addition, all of them provided written consent through a protocol reviewed by the the research teams of the Laboratoire IMS and the UMA-BCI group.

### 2.2. Data Acquisition and Signal Processing

The electroencephalographic (EEG) signals were recorded using the following electrode positions: Fz, Cz, Pz, Oz, P3, P4, PO7, and PO8, according to the 10–20 international system. All channels were referenced to the left earlobe, using Fpz as the ground. The signal was amplified through a sixteen-channel biosignal amplifier (gUSBamp, Guger Technologies, Schiedberg, Austria). The amplifier settings were 0.5–100 Hz for the band-pass filter, the notch (50 Hz) was on, and the sensitivity was 500 μV. The signal was then digitized at a rate of 256 Hz. EEG data collection and processing were controlled by BCI2000 software [31]. A stepwise linear discriminant analysis (SWLDA) of the data was performed to obtain the weights for the P300 classifier and to calculate the accuracy (using the BCI2000 tool called *P300Classifier*). A detailed explanation of the SWLDA algorithm can be found in the *P300Classifier* user reference [32].

### 2.3. Experimental Conditions

Three different experimental conditions, based on a similar paradigm, were tested in the present work. The common factor in all conditions was that users had to attend to a specific auditory stimulus. Four different tones were used as stimuli: a beep at 200 Hz in the left earphone, a beep at 1000 Hz in the left earphone, a beep at 200 Hz in the right earphone, and a beep at 1000 Hz in the right earphone. These frequencies (tones) have been previously used elsewhere [33] in an auditory BCI. In all conditions, users had to attend to a designated target stimulus and ignore the other stimuli (primary task). When the target stimulus appeared, subjects were asked to perform a specific mental task. All stimuli were presented for 300 ms with an inter-stimulus interval (ISI) of 363 ms. Thus, the stimulus onset asynchrony (SOA) was equal to 663 ms. The four auditory stimuli were delivered, and randomly distributed, in each sequence (4 stimuli per sequence, that is, 2.65 s). Each run contained a number of sequences, which were variable according to the subject and the type of session. The specific mental task asked of the participant was to mentally count how many times, in a run, the target stimulus occurred in order to produce the P300 component in the oddball paradigm.

The study compared three conditions: (i) a control condition (C1) that consisted of an ERP-based auditory BCI with no audio background, (ii) a second condition (C2) based on the control condition while simultaneously playing an audio background that the participant had to ignore (non-attentional speech), and (iii) a third condition (C3) based on the second condition while the participant had to pay attention (secondary task) to the audio background (attentional speech). Each subject participated in offline and online sessions for the three conditions.

The background speeches used as passive and active distractors (conditions C2 and C3, respectively) were extracted from didactic material for advanced-level French language students (level B2-C1). These audios presented a TV news format with explanations of various topics and interviews. The volume level of the BCI (the tones) and the background audio (the speeches) was similar for all participants and conditions. The background audios were counterbalanced to be equally presented for C2 and C3 conditions.

**Figure 1:**
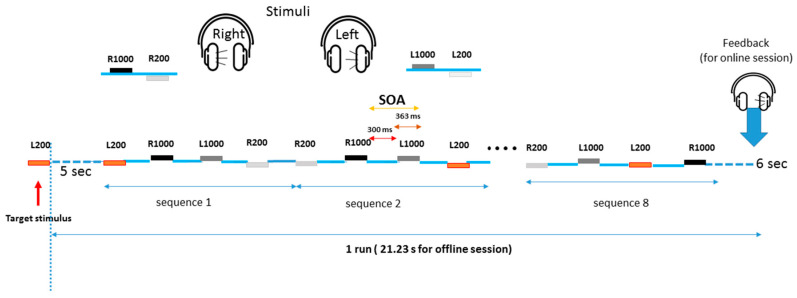
Trial time of a run. The number of sequences was 8 for the offline sessions and variable for the online sessions. The stimuli are R1000 (1000 Hz right), R200 (200 Hz right), L1000 (1000 Hz left), and L200 (200 Hz left). In this illustration, the target stimulus is presented in brown (200 Hz Left in this example). The feedback was only provided in the online sessions.

In the offline session, each run contained eight sequences. At the beginning of each run, the stimulus target was presented to instruct the user on which stimulus to mentally count. The sequences started 5 s after the presentation of the target. There was a 6 s break at the end of each run, and no feedback was provided. Thus, a run for the offline session took 21.23 s (5 s × 4 stimuli × 8 sequences + 6 s). For a schematic illustration of a run, see Figure 1.

In the online session, the trial time of a run was the same as that of the offline session, however, the number of sequences per run was adapted for each subject and condition according to the ITR (information transfer rate) obtained after analyzing the EEG signals recorded during the offline session. At the end of the run, the tone that the BCI system identified was presented to the subject for 0.5 s.

### 2.4. Procedure

The experiment was carried out in an isolated room. The participants were seated in a comfortable chair. Conventional headphones were used to present the auditory stimuli. A within-subject design was used so all users went through all experimental conditions. The study was performed in a single session lasting approximately 80–100 min. The order of the condition’s presentation was selected pseudo-randomly following a complete block design. Participants were instructed in order to perform the task correctly. In all conditions, they were asked to focus their attention on the target stimulus. For C2, participants were instructed to ignore the background speech that was designed to distract them. Finally, for C3, participants were instructed to attend to and retain the information in the background audio while performing the BCI task. For both C2 and C3, participants were aware that they would later be asked certain questions regarding the background speech. Participants were also recommended to perform the task with their eyes closed as this has been shown to improve the usability of the BCI [34].

All conditions consisted of two parts. The first part, the offline session, was an initial calibration task to obtain the specific user’s brain signal parameters and to adapt the system to the user. The signal processing carried out with the data obtained in this calibration session was detailed in the section on data acquisition and signal processing. The second part was the online task in which the user received feedback according to the stimulus selected. 

Each condition consisted of three blocks for the calibration task and two blocks for the online task. In each block, the user had to perform eight runs, that is, eight selections. Then, a total of 24 selections were performed in the calibration task, and 16 in the online task. The specific order for the target designated for selection was the same for each block: left 200 Hz, left 1000 Hz, right 200 Hz, right 1000 Hz, left 200 Hz, left 1000 Hz, right 200 Hz, and right 1000 Hz. A short break between blocks (variable at user request) was employed.

**Figure 2:**
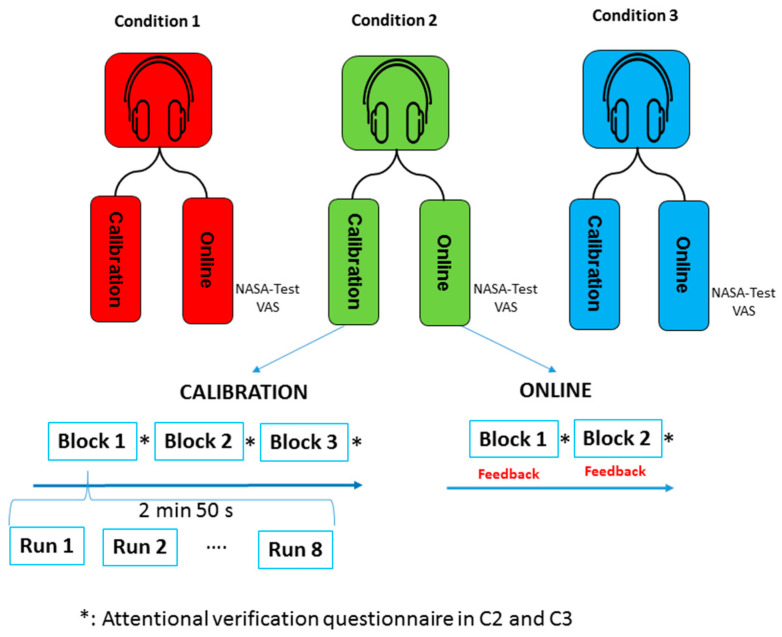
Experimental protocol. Each condition consisted of three calibration and two online blocks. Each block consisted of 8 runs (2 min 50 s for the calibration sessions, and shorter for the online sessions). The attentional verification questionnaires were provided at the end of each block only for conditions C2 and C3.

At the end of each block, the user had to complete an attentional verification questionnaire to check that their attention to the background speech (secondary task) was higher in C3 than in C2. After each condition, participants were asked to complete a visual analogue scale (VAS) of fatigue and the NASA-TLX questionnaire in relation to the induced workload during the experiment. A schematic illustration of the experimental protocol is shown in Figure 2.

### 2.5. Information Transfer Rate (ITR)

The information transfer rate (ITR) [35] is the number of bits transmitted per run. The ITR allows the establishment of an objective measure that considers, besides the accuracy, the number of elements available in the interface and the time to select an element:

(1) $$B=\;\log_{2}N+P\log_{2}P+\left(1-P\right)\log_{2}\frac{1-P}{N-1}$$

(2) $$\mathrm{ITR}=\{\begin{array}{c} \frac{B}{T},\;\;P>0.25\\ 0,\;\;P\leq 0.25 \end{array}$$

with *B* being the number of bits of information contained in each selection, *P* being the accuracy of correct classification, *N* being the number of elements available in the interface (*N* = 4), and *T* being the time needed to complete a run. It is worth mentioning why we fixed the ITR to zero if *p* < 0.25: when the accuracy result is lower than 25%, the calculation considers that it still provides some bits of information, even when it is erroneous information. It was decided that under the threshold of chance (25%) no information could be considered useful when comparing experimental conditions.

Since the SWLDA algorithm provided the accuracy at each sequence, it is possible to calculate the resulted ITR in the calibration session at each sequence.

### 2.6. Evaluation

#### 2.6.1. Performance

To evaluate the performance on the calibration phase, the accuracy of the system to classify the selections was calculated for each sequence. The ITR was also obtained in order to determine, for each subject and condition, the number of sequences to use during the online session, being the number of sequences selected that reach the highest ITR.

For the online task, the accuracy and the ITR were employed. In contrast to the calibration task, the accuracy for the online task was calculated by just dividing the number of correct selections by the total number of selections made (two blocks of eight selections). This *accuracy* was used for the calculation of the online ITR, considering the time *T* to complete a run according to the number of sequences used.

#### 2.6.2. P300 Component

The P300 component is a positive deflection produced by the perception of an infrequent expected stimulus. The maximum amplitude is usually reached on central to parietal regions between 250 and 500 ms. However, the auditory P300 has shown a larger latency than that of the visual modality in BCI [36]. In order to proceed with the statistical analysis for P300 and to study how it may be affected under different conditions, a topographical analysis was carried out. The specific time intervals for this component were chosen based on the specific results of previous works on an auditory ERP-BCI (e.g., Huang et al. [33], Hübner et al. [34] and Onishi et al. [37]). The program used for the analysis of the EEG signal was EEGLAB (v13.6.5b) [38]. One participant was eliminated for waveform ERP analysis because his/her signal was too noisy to be included without altering the grand average. However, his/her results related to performance and workload were considered in the analyses as they were not affected.

#### 2.6.3. Cognitive Workload and Fatigue

In order to evaluate the effect of passive or active auditory distractors on BCI user experience, the NASA-TLX test [39] and a visual analogue scale (VAS) related to fatigue were used. On one hand, the NASA-TLX produces a total workload score (from 0 (very low) to 100 (very high)) calculated from six subscales: mental demand, physical demand, temporal demand, effort, performance, and frustration. The total workload was computed by the weighting average technique, which considers the particular contribution of every subscale; the scores of the subscales were those directly selected by the participants (0–100 in intervals of five points). On the other hand, the VAS fatigue ranged between 0 (very low) and 10 (very high), and the user simply had to score the level of fatigue felt after controlling the corresponding condition.

#### 2.6.4. Attentional Verification to Secondary Task

To verify whether the participant had paid attention to the background audio according to the instructions in C2 (passive listening) and C3 (active listening), a written questionnaire of 5 questions was presented at the end of each block. For each condition, 25 three-choice questions were presented about the content of the audios. Participants had to answer all the questions. The calculated score (i.e., the dependent variable) was the percentage of correct answers.

#### 2.6.5. Statistical Analysis 

To analyze performance (i.e., accuracy and ITR) and workload, ANOVA was used to examine if there were significant differences between conditions. The Greenhouse-Geisser correction was used to check for any violation of the sphericity assumption [40]. When significant effects were found in the ANOVA, multiple post hoc comparisons between specific conditions were made using Holm’s correction method.

For the analysis of the ERP waveform, the paired *t*-test was applied, comparing the amplitude (µV) obtained in all channels between (i) the target versus non-target stimuli of each condition and (ii) pairs of conditions for each stimulus (target and non-target). The paired *t*-test analyses related to the ERP waveform were also corrected using Holm’s method, because multiple channels were compared simultaneously.

Finally, for the attentional verification test, a paired *t*-test was employed to check whether there was a significant difference between conditions C2 and C3 with regard to the amount of information retrieved from the audios.

The statistical analyses of performance, workload, and attentional verification were carried out using JASP software [41]. The analysis of the ERP waveform was carried out with EEGLAB [38]. The standard error was the measure of variance used to present the results.

## 3. Results

### 3.1. Performance Analysis

#### 3.1.1. Calibration Task

**Figure 3:**
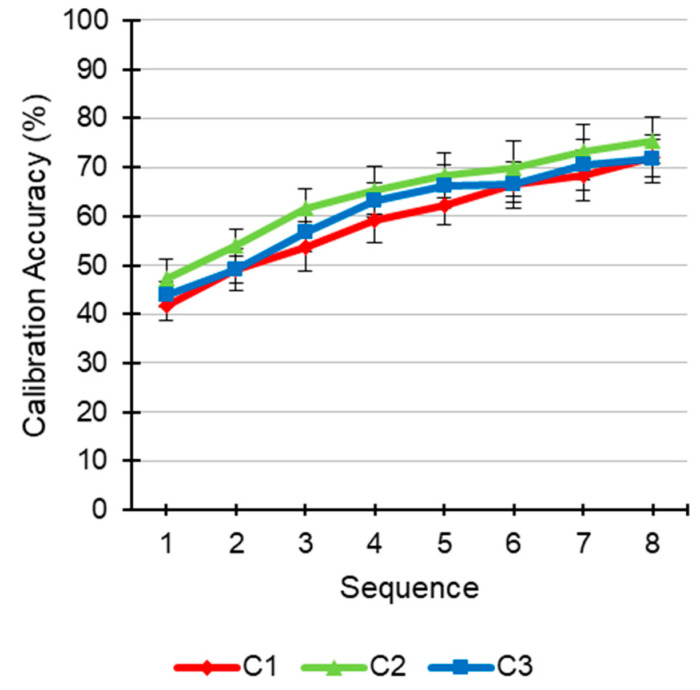
Accuracy (%; mean ± standard error) of each condition (C1, no speech; C2, ignoring the speech; C3, listening to the speech) as a function of the number of sequences during the calibration phase.

Figure 3 shows the averages of the classification accuracy in the calibration task achieved by the participants under the different conditions as a function of the number of sequences. The majority of the participants presented a similar accuracy, reaching values above 70%. A two-way repeated-measures ANOVA (3 × 8) including the condition (C1, C2, and C3) and sequence (eight sequences length) as factors was carried out with accuracy as dependent variable. The sequence factor offered significant differences (*F* (7, 70) = 36.406, *p* < 0.001, *η*^2^ = 0.31). Neither the condition factor (*F* (2, 20) = 0.637, *p* = 0.539, *η*^2^ = 0.017) nor the condition × sequence interaction (*F* (14, 140) = 0.238, *p* = 0.998, *η*^2^ = 0.002) were significant.

#### 3.1.2. Online Task

**Figure 4:**
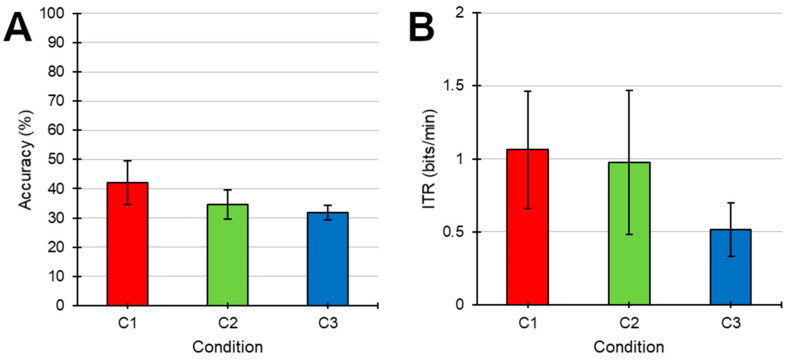
**(A)** Accuracy (%; mean ± standard error) and (**B**)ITR (bits/min; mean ± standard error) of each condition (C1, no speech; C2, ignoring the speech; C3, listening to the speech) during the online phase.

Figure 4 shows the overall online classification accuracy and ITR for each condition. The average values regarding the number of sequences required to select a stimulus, the accuracy, and the ITR obtained for different conditions were as follows: number of sequences: C1, 4.27 ± 0.63; C2, 3.82 ± 0.74; C3, 3.09 ± 0.72 (F (2, 20) = 1.479; *p* = 0.252; η^2^ = 0.046); accuracy: C1, 42.05% ± 7.49%; C2, 34.66% ± 5.01%; C3, 31.82% ± 2.45% (*F* (2, 20) = 1.365; *p* = 0.278; *η*^2^ = 0.06); ITR: C1, 1.06 ± 0.4 bits/min; C2, 0.98 ± 0.49 bits/min; C3, 0.52 ± 0.18 bits/min (*F* (2, 20) = 0.853; *p* = 0.441; *η*^2^ = 0.038). Although these variables did not show significant differences between conditions, a tendency for a decreased ITR in the presence of distractors with attentional task was observed (C3).

### 3.2. P300 Component Analysis

**Figure 5:**
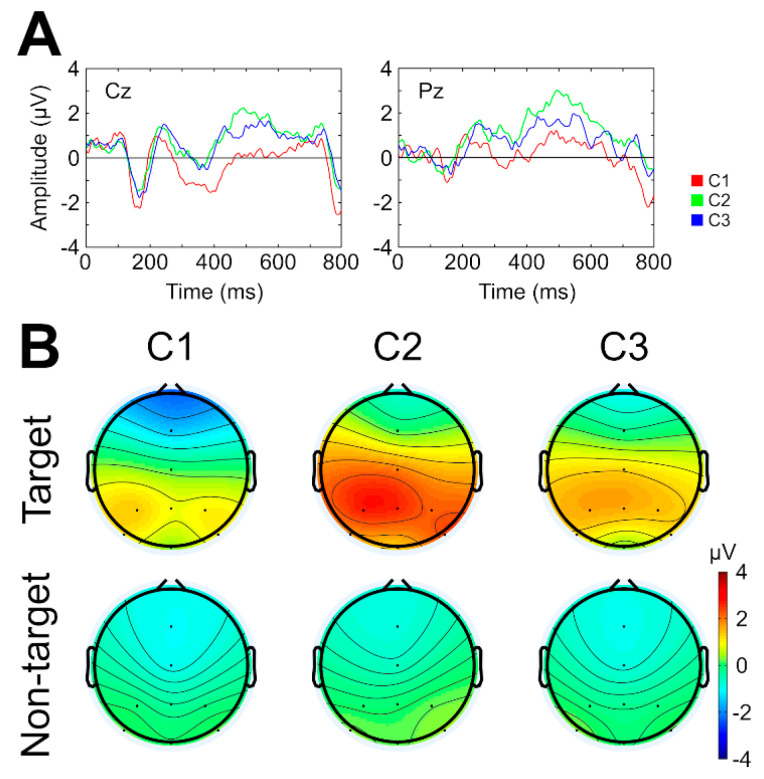
(**A**) Grand average event-related potential (ERP) waveform (microvolts) for target stimuli in Cz and Pz, for the three conditions (C1, no speech; C2, ignoring the speech; C3, listening to the speech). (**B**) Topographical scalp map for the P300 component (450–550 ms) of each condition. Both plots have been obtained from the calibration phase.

Figure 5, pane A shows, as examples, the average evoked potentials of the two electrodes from the central and parietal regions (Cz and Pz). As can be observed, the peak for the auditory P300 was elicited around 450–550 ms. Therefore, this interval was chosen to perform the topographic analysis and to search for significant differences in the amplitude of these components according to the paired *t*-test. Significant differences were found between the conditions in the amplitude levels of component P300 (Figure 5, panel B). Specifically, for the target stimuli, a significantly higher amplitude at C2 versus C1 in several channels was observed (*p* = 0.028 for Fz, Cz, and Pz; *p* = 0.032 for P3, and *p* = 0.049 for P4, all these post hoc analyses were performed using the Holm’s correction method). In addition, between target and non-target stimuli, the analysis showed a significantly higher P300 amplitude level for target stimuli for condition C2 in Cz, Pz, and P3 channels (*p* = 0.04 for each channel, using the Holm’s correction method).

### 3.3. Cognitive Workload and Fatigue Analysis 

**Figure 6:**
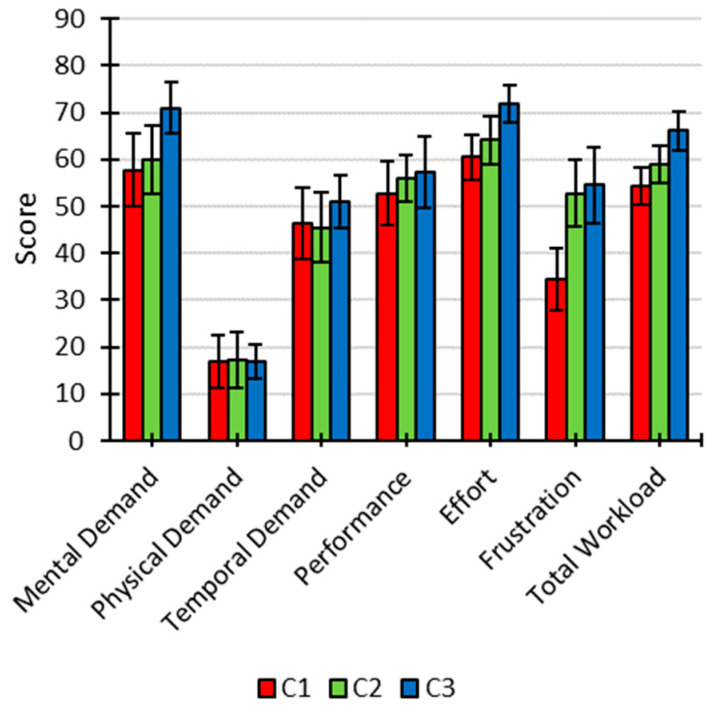
Scores (mean ± standard error) of total workload and unweighted six subdimensions for each condition (C1, no speech; C2, ignoring the speech; C3, attending to the speech) for NASA-TLX.

Figure 6 shows the overall contribution of the total workload and dimensions to assess the subjective workload for each condition. The variables related to NASA-TLX that offered significant differences according to the condition employed were as follows: total workload (C1, 54.33 ± 4.01; C2, 58.88 ± 4.04; C3, 66.06 ± 4.19 (*F* (2, 20) = 5.082; *p* = 0.016; *η*^2^ = 0.134)), mental demand (C1, 57.73 ± 7.89; C2, 60 ± 7.35; C3, 70.9 ± 5.42 (*F* (2, 20) = 4.769; *p* = 0.02; *η*^2^ = 0.07)), and frustration (C1, 34.55 ± 6.61; C2, 52.73 ± 7.19; C3, 54.55 ± 8.11 (*F* (2, 20) = 4.738; *p* = 0.02; *η*^2^ = 0.143)). Next, post hoc analyses were performed, using the Holm’s correction method, to identify between which specific conditions there were significant differences. For total workload, it was found that the score obtained by C3 was significantly higher than that of C1 (*p* = 0.014; *d* = 1.091). On the other hand, for mental demand, a significantly higher score was observed for C3 compared with C1 (*p* = 0.037; *d* = 0.918) and C2 (*p* = 0.037; *d* = 0.865). Regarding frustration, it did not show any significant differences in the multiple comparisons. However, when Holm’s correction method for multiple comparisons was not applied, the frustration score of C1 was significantly lower than that of C2 (*p* = 0.049; *d* = 0.675) and C3 (*p* = 0.023, *d* = 0.81). The VAS fatigue did not show significant differences according to the condition (C1, 5.09 ± 0.65; C2, 4.82 ± 0.54; C3, 4.55 ± 0.68 (*F* (2, 20) = 0.338; *p* = 0.717; *η*^2^ = 0.013)).

### 3.4. Attentional Verification to Secondary Task

The control questionnaire showed significant differences between the percentage of correct answers of C2 and C3, in favor of C3 (C2, 52.73% ± 6.88%; C3, 69.09% ± 3.37% (*t* (10) = 2.72; *p* = 0.022; *d* = 0.82)). Thus, it can be confirmed that the participants followed the instructions correctly and paid more attention to the background speech in condition C3 than in C2.

## 4. Discussion

The aim of the present study was to assess the impact, in an auditory BCI, of a background speech with passive and active attention, on performance, ERP waveform, and workload. There were significant increases in P300-ERP signal for the passive attention condition, and it was more important for targets than for non-targets. In contrast, the active attention condition, in which participants were instructed to pay attention to the background speech, elicited rather important increases in cognitive workload without producing changes in P300 signal compared with the control condition, that is, without distractors. The results indicate that listening to the speech significantly impaired the user experience in an auditory BCI. This effect was specifically observed through an increase in some of the workload measures (total workload, mental demand, and frustration). 

The novelty of this study was twofold: (i) there is no previous study that has evaluated the effect of a background speech in an auditory ERP-BCI and (ii) it was based on the use of two control conditions (C1 and C2): one to study the simple presence of the background audio (C1 vs. C2 and C3), and a second to study the effect of the secondary task of attention (C2 vs. C3). This complete control design was not used in previous studies involving distractors in an ERP-BCI (i.e., Käthner et al. [15] or Xu et al. [16], who used a visual ERP-BCI, and Zhou et al. [17], who used an auditory ERP-BCI). First, Käthner et al. [15] lacked control conditions for the presence of audio (i.e., the condition C1). Secondly, Xu et al. [16] also did not evaluate the presence of audio or a secondary attentional task as it did not have a condition without audio or with audio, but no attention (i.e., C1 or C2, respectively). Finally, Zhou et al. [17] did not present a condition with attention (i.e., C3). 

In the present work, the secondary attentional task (i.e., to listen to the speech) has been correlated with workload. From a cognitive point of view, it suggests that listening to a background in an auditory ERP-BCI is a highly demanding task that may involve additional attentional resources. Specifically, the addition of a speech with attention (C3) led to a significant increase in total workload compared with the condition without speech (C1) and significantly increased the mental demand experienced by the user, compared with the absence of speech (C1) or even passive listening (C2). Finally, although not significant in the multiple comparisons (without Holm’s correction significant results were obtained), the mere presence of speech, with or without attention (C3 and C2, respectively), led to increased frustration. In summary, using an auditory ERP-BCI, we show that a background speech produced a greater cognitive workload for the attentional condition (C3). This observation is similar to those obtained by Käthner et al. [15], who evaluated the effect of background audio stories in a visual ERP-BCI and concluded that the workload was negatively related to listening to the background audio story. In addition, while Zhou et al. [17] observed that the background music improved the user experience, the present work has shown that the simple addition of a background speech (C2) did not produce any positive significant effect on subjective measures. Therefore, it suggests that the background sounds might produce different effects depending on the particular type of auditory stimulus.

Regarding performance, it is known that auditory ERP-BCI usually presents a lower accuracy compared with visual ERP-BCI, and it was observed equally in this study for both accuracy and ITR values [42]. Despite the effect on workload, there were no significant differences between conditions in terms of performance. However, in the online phase, a tendency to decrease performance was observed in the presence of audio distractors with the active attentional task (C3). This tendency of C3 to offer lower performance could be related to its higher workload scores. These results are consistent with those obtained by Käthner et al. [15] and Xu et al. [16], who found a negative link between performance and workload. However, because neither of the differences in performance were significant, as was the case in Zhou et al.’s study [17]—the only one that has evaluated the effect of auditory distractors on an auditory ERP-BCI—it may be suggested that auditory distractors have a lower effect on performance than that shown in visually based ERP-BCI proposals [15,16]. Therefore, the effect of the distractors in the same modality has not been observed to produce greater interference with performance than in a different sensory modality using a visual ERP-BCI and auditory distractors [15,16]. Further work would be required to assess this possible differential effect according to the ERP-BCI modality. 

Concerning the P300 component, in the present work, it has been observed that brain activation at 450–550 ms post-stimulation was significantly higher for target stimuli in central and parietal regions (electrodes Fz, Cz, Pz, P3, and P4) in the presence of non-attended distractors (C2), compared with control condition without distractors (C1). These results suggest that when participants are instructed to ignore distractors, irrelevant stimuli like speech background should not produce interference, allowing higher selective attention on target stimuli of the primary task. Furthermore, a decrease in P300 amplitude for the target stimuli was not observed to be significantly related with a higher workload (the P300 amplitude in C3 did not show significant differences versus C1 or C2), as was observed by Käthner et al. [15] and Xu et al. [16]. This might suggest that, in C3, two opposite attentional effects are co-occurring in the same sensory modality (auditory) on the P300 component: (i) a positive effect (increased P300) caused by the addition of the background speech versus (ii) a negative effect (decreased P300) produced by the secondary attentional task that demanded high workload. Effectively, the two previous papers, which assessed the effect of auditory distractors on a visual ERP-BCI, showed that workload and attention to background audio decreased the amplitude of the P300 component [15,16]. Specifically, Käthner et al. [15] noted a significantly lower amplitude of the P300 for the attentional condition (requiring higher workload) than for the non-attentional condition. In our study, in spite of lack of significance, similar results are observed in Figure 5 (Panel B); there was a tendency for the P300 to be lower in C3 than in C2. One cause of this lack of significant difference could be that workload on the attentional condition (C3) was lower than that obtained in Käthner et al. [15], without presenting the sufficient negative effect expected in the P300 that would provoke significant results. Likewise, the possibility that the effect of the workload or the P300 may be different as a result of the type of BCI modality (visual or auditory) or due to other parameters (e.g., number of stimuli or presentation times such as stimulus duration or SOA) cannot be discounted. 

Zhou et al. [17]—in the only previous study that has evaluated the effect of background sounds in an auditory ERP-BCI—did not carry out the pertinent ERP analyses in their study, but, after analyzing their ERP waveform figures, there seems to be no difference between the conditions with and without background music. However, according to our results, the presence of a background speech to be ignored increases significantly the P300 amplitude for target stimuli. It is possible that the larger P300 amplitude of C2 was a result of the interrelationship between the audio background and the BCI interface. The probability of a target stimulus occurrence in an oddball paradigm affects the amplitude of the P300: the lower the probability, the higher the amplitude of the P300 [43]. In the present study, the target stimulus was presented 25% of the time (one target, three non-target). However, in the C2 condition, the background audio stimuli (for example, the different words pronounced during speech) may have acted as non-target stimuli. In this condition, even though this background was not supposed to have been attended to, users answered correctly 52.73% of the questions asked in the control questionnaire. In this sense, the ratio of target stimuli to non-target could have been much lower than the 25%, increasing the amplitude of the P300 compared with C1 condition. This effect may have also occurred in C3 but, in this C3 condition, the subjective workload was higher because attentional resources were divided between two unimodal tasks, which consequently may have reduced the amplitude of the P300 in comparison with C2.

The main limitations in the present study were the low performance in the online phase and the high variability in that variable among participants. On one hand, the low performance in the online phase could be explained by the much more exigent criterion used to decide the number of sequences used in the online phase, which was based on the maximum ITR. On the other hand, in order to avoid the high variability in performance, which could make it difficult to obtain statistically significant differences, future experiments should aim to minimize or control this variability either by increasing the sample size (in order to reduce the standard error) or by making the sample more homogeneous through certain criteria or covariance analysis, using questionnaires on variables that have been shown to influence performance, such as emotional stability or stress [44,45]. However, despite the above-mentioned limitations, we would argue that our results are clear in two respects: (i) workload is affected when attention is paid to external speech and (ii) there is an increase in the P300 amplitude of the target stimulus because of the presence of background speech that the user should ignore. These effects could be considered by future researchers, so that the user experience in an auditory ERP-BCI under disruptive environments might be enhanced.

## 5. Conclusions

The aim of this work was to assess the impact of an audio distractor (background speech) in an auditory ERP-BCI. Results showed that distractors impinged the user experience through a negative effect on cognitive workload (total workload and mental demand), which increased significantly when external audios were attended to (secondary task) simultaneously with the control of the BCI (primary task). In addition, our results suggest that the performance could be partially affected by the presence of a distracting audio that implies an attentional shift, particularly in longer sessions or in those using more complex audios where the workload could be increased further. Furthermore, the analysis of the P300 component showed that the addition of background audio without attention (C2) significantly increased the amplitude of the target stimuli in several channels compared with the absence of such audio (C1). 

The present study has proven that background speech, especially when it is attended to, is an important interference that should be avoided while using an ERP-BCI. Therefore, the inevitable presence of background speech in the real world might present a problem for real-world BCI applications. Future work could explore the design of interfaces that avoid the negative effect of workload on the user experience produced by attention or the mere presence of an external audio. Some possible solutions could include bypassing the external sound through noise cancellation or integrating it into the BCI control and using it to allow the user to better focus their attention on the desired stimulus/command (i.e., using external audio as the BCI control stimulus). Also, the increase in amplitude of the target stimulus for the passive audio condition (C2) might be studied further to improve the performance of the P300 classifier.

## Data Availability

Due to privacy restrictions, data presented in this study are available on request from the corresponding author.
